# High expression level of homocitrulline is correlated with seborrheic keratosis and skin aging^⋆^

**DOI:** 10.1016/j.abd.2022.07.002

**Published:** 2023-01-05

**Authors:** Juping Chen, Jun Liu, Zheng Wang, Jiandan Xu, Jia Tao, Hualing Li

**Affiliations:** a Department of Dermatology, Affiliated Hospital of Yangzhou University, Yangzhou, PR China; b Institute of Translational Medicine, Medical College, Yangzhou University, Yangzhou, PR China; c Department of Pathology, Affiliated Hospital of Yangzhou University, Yangzhou, PR China; d Jiangsu Key laboratory of Experimental and Translational Non-Coding RNA Research, Yangzhou, PR China; e Jiangsu Co-Innovation Center for Prevention and Control of Important Animal Infectious Diseases and Zoonoses, Yangzhou, China

**Keywords:** Protein carbamylation, Homocitrulline, Skin aging, Seborrheic keratosis

## Abstract

**Backgroud:**

Homocitrulline (Hcit), is involved in the pathological processes of some diseases. However, the role and function of Hcit (CBL) in human skin remains largely obscure.

**Objective:**

To investigate the correlation of the level of Hcit in seborrheic keratosis, skin aging, and its clinical significance.

**Methods:**

Immunohistochemistry was used to analyze the level of Hcit in skin lesions of seborrheic keratosis (SK), unaffected skin (distant 0.5 centimeters from SK lesion), and normal skin of healthy subjects in the control group. ELISA test was used to detect the serum level of CBL in SK patients and healthy subjects of different ages.

**Results:**

Hcit was mainly localized in the nucleus of epidermal cells. In healthy control skin, the expression of Hcit increased with age and showed a positive correlation with age (the correlation coefficient was 0.806, p = 0.0002). The expressional level of Hcit in SK lesions was higher than that in healthy control skin (Z = −3.703, p = 0.0002). The serum level of CBL in healthy subjects and in SK patients increased with age (the correlation coefficient were 0.5763, p = 0.0032; 0.682, p = 0.004. respectively). The serum level of CBL in SK patients was higher than that in healthy subjects (Z = −2.19, p = 0.030).

**Study limitations:**

The small serum sample size in the study.

**Conclusion:**

The high expressional level of Hcit is correlated with seborrheic keratosis and skin aging. HCit may be one of the potential biomarkers of skin aging.

## Introduction

Skin aging is a long and complex process, and it is an external manifestation of aging.^1^ Scientists have developed SCINEXA, which is a clinical scoring system for skin aging. It evaluates skin aging on the basis of 5 indexes: uneven pigmentation, skin wrinkles, sagging skin, reduced adipose tissues, and benign skin tumors.^2^ Seborrheic Keratosis (SK) is the most common benign skin tumor, and it is also one of the conditions that indicate skin aging. In general, SK is also known as basal cell papilloma and is a benign intraepidermal tumor, which is caused by a delay in the maturation of keratinocytes.^3^ The incidence of SK increases along with increasing age.^4^

With an increase in age, the skin-resident immune cells gradually undergo aging, which is a process that weakens the repairability of the skin tissue. Moreover, external factors such as ultraviolet radiation, and air pollution, and internal factors such as diabetes and vascular diseases further accelerate the aging of skin tissues. During the life cycle of an organism, proteins are exposed to various metabolic stresses, causing a gradual change in the structural and functional properties of skin tissues. This phenomenon is mainly triggered by non-enzymatic post-Translational Protein Modifications (nePTMs), such as glycosylation and carbamylation reactions.^5^ The carbamylation reaction is a process in which cyanate and its structural isomer, that is, isocyanate, combine with free functional groups of proteins, peptides, or free amino acids.^6^ In carbamylation reaction, different functional groups interact and produce different carbamylated derivatives, this reaction preferentially occurs on the ε-amino group of lysine and forms N(6)-Carbamoyl-L-Lysine (CBL), which is commonly known as Homocitrulline (HCit)^7^: it is the most characteristic derived product of protein carbamylation reaction. Previous studies have reported that protein carbamylation reaction is involved in pathological processes of chronic diseases, such as chronic kidney disease, atherosclerosis, and rheumatoid arthritis.^8^, ^9^, ^10^ On the other hand, very few studies have investigated the role of protein carbamylation reaction in the skin aging process. Gorisse et al.^11^ quantitatively analyzed the contents of HCit in total skin extract, and they isolated type Ⅰ collagen proteins from human, cow, and mouse skin tissues. They found that the concentration of HCit increases significantly with an increase in age. They performed an immunofluorescence assay of the epidermis and dermis of the skin, which was obtained from 20-and 80-year-old subjects, the results indicate that in the skin tissues of 80-year-old subjects, the epidermal and dermal type I collagen proteins exhibit a stronger fluorescence response, show more deposition of carbamylated proteins in this group.

Carracedo et al.^12^ analyzed carbamylated proteins in the peripheral blood of healthy subjects. They found that in the elderly group (60‒79-year-old), the carbamylated protein level was higher than that in the young group (20‒39-year-old) and middle-aged group (40‒59-year-old). All these results indicate that there is a correlation between HCit, age, and senescence. SK is a very common disease in dermatology, some studies have reported that SK occurs due to the mutation of Fibroblast Growth Factor 3 (FGFR3).^13^, ^14^ Christian Hafner et al found that 57% of SK patients had FGFR3 mutation, moreover, FGFR3 mutation was found to be significantly associated with increased age, and the frequencies of FGFR3 mutation were significantly higher in the head and neck region but lesser in the trunk and limbs of patients. This indicates that SK occurs due to UV exposure and local skin aging. Furthermore, SK is mostly correlated with age.^4^ Alzheimer’s disease is a manifestation of aging in human beings. Kang M J et al. have found that Amyloid Precursor Proteins (APPs) are related to Alzheimer’s disease. They reached a consensus that “senile plaque” in the brain is a special marker of Alzheimer’s disease, and this “senile plaque” is formed due to the accumulation of β-amyloid protein (a downstream product), which is produced by sequential proteolytic cleavage of APP.^15^ In a study conducted by Li Y et al., APPs were greater in SK patients and increased with the age of patients.^16^ These above studies show that SK is not only a manifestation of skin aging, but also related to human aging. Presently, aging has many manifestations and detection methods, but it does not have any special objective indicator for laboratory evaluation. In order to provide more favorable evidence and to establish the correlation between HCit and skin aging, the authors investigate the different expressional levels of HCit in SK patients and healthy control skin to further determine whether Hcit can reflect the difference between aging and non-aging skin. The authors also determined whether there was a variation in serum level of CBL between healthy subjects and SK patients, the authors further compared the serum expressional levels of CBL among healthy subjects and SK patients of different ages on the basis of the predecessors.

## Materials and methods

### Collection of clinical specimens

Inclusion criteria: This study was conducted between September 2018 and June 2020. The skin lesion and unaffected skin in SK patients, which was confirmed by pathological biopsy, were treated in the Dermatology of Affiliated Hospital of Yangzhou University, Yangzhou, Jiangsu, China. The healthy control skin (without any skin disease) tissues of different ages were collected from the Department of Plastic Surgery of Affiliated Hospital of Yangzhou University, Yangzhou, Jiangsu, China. All samples were treated by the same dermatologist. In the skin tissues, there were 19 SK patients in the experimental group, their ages ranged from 41 to 82 (61.4 ± 13.6) years, on the other hand, there were 16 healthy subjects in the control group, their ages ranged from 23 to 82 (50.2 ± 17.7) years. The skin lesion, unaffected skin in SK patients and the healthy control skin of different ages were all breast skins harvested from male subjects. The unaffected skin was collected 0.5 centimeters from SK skin. All serum samples were collected from the Department of Dermatology and Physical Examination Center of the Affiliated Hospital of Yangzhou University, Yangzhou, Jiangsu, China. Serum samples were collected from males, among them, 16 were healthy subjects: 8 of them were in the age group of 30–49 years, and their average age was 39.5 ± 6.3 years. 8 subjects were over 60 years old, and their average age was 74.25 ± 6.27 years. In 16 SK patients of serum samples, 8 were in the age group of 30‒49 years, their average age was 38.5 ± 5.15 years. The remaining 8 were over 60 years old, and their average age was 70.6 ± 7.85 years (Table 1).

**Table 1:** The demographic data of the studied groups.

	Healthy subjects		SK patients	
	Skin	Serum	Skin	Sreum
**n**	16	16	19	16
**Age (years)**				
**Mean (SD)**	61.4 (13.6)	56.9 (18.2)	50.2 (17.7)	54.6 (17.1)
**Sex, n (%)**				
**Male**	16 (100%)	16 (100%)	19 (100%)	16 (100%)
**Female**	0 (0)	0 (0)	0 (0)	0 (0)
**Skin collected region**	Breast		Breast	
**Photodamaged**	Not		Not	

The skin and serum samples were not collected from the same individuals.

SK, Seborrheic Keratosis.

Exclusion criteria: Patients with chronic diseases, such as hypertension, diabetes, and heart diseases were excluded from this study. Moreover, patients with other diseases such as kidney disease, autoimmune diseases, or malignant tumors were also excluded. The experimental protocol was approved by the Medical Ethics Committee of the Affiliated Hospital of Yangzhou University, Jiangsu, China (approval Nº NO2018525). All the samples were collected from patients who signed an informed consent letter.

### Immunohistochemistry analysis

After collecting skin tissues, the authors embedded them in paraffin and cut them into sections. Then, these sections were subjected to several processes, such as deparaffinization, dehydration, antigen repair, endogenous peroxidase blocking, serum blocking, the primary antibody of HCit (rabbit anti-human polyclonal antibody, Covalab Company, (Bron, Rhone-Alpes Region, France), the working concentration of HCit antibody was 1:60. Goat anti-rabbit secondary antibody (Beijing Zhongshan Goldenbridge Biotechnology Co. Ltd., Beijing, China), 3.3’-Diaminobenzidine (DAB) color development, hematoxylin restaining, dehydration, transparentizing, and mounting on glass slides for observation. Finally, the images were obtained, and the expressional level of HCit was analyzed by 2 dermatopathologists. The immunohistochemical expression of HCit was observed under a low-power microscope (Olympus Corporation, Tokyo, Japan): 10 high-power microscope fields (400×) were randomly selected for observing each section, and scoring was performed as per staining intensity and the percentage of positive cells in the epidermis. The percentage of positive cells was scored as 0‒4 points: < 5%, 0; point: 5%‒25%, 1; point: 25%‒50%, 2; points: 50‒75%, 3; points: ≥ 75%, 4 points. The staining intensity was scored as 0‒3 points: non-staining 0, Light yellow 1, Brownish yellow 2, Chocolate brown 3. The above-mentioned points were multiplied together to obtain the final score, and the higher the score, the stronger the positive expression of HCit.

### Enzyme-linked immunosorbent assay (ELISA)

Hcit is a kind of protein carbamylated derivatives, which preferentially occurs on the ε-amino group of lysine and forms N(6)-carbamoyl-L-lysine,^7^ it is also called Carbamyl-Lysine (CBL). The quantity of CBL of protein samples can represent the level of carbamylation of proteins, which is commonly known as Hcit. Blood samples were collected from healthy subjects and SK patients of different ages. After centrifuging the blood samples, the supernatants were stored in a refrigerator at −80 °C or directly used in the next experiment. The testing sample and CBL-BSA standard were added into a plate, which was coated with an anti-CBL antibody (Carbamyl-Lysine) (Cell Biolabs Corporation, San Diego, California, USA), this plate was incubated at 37 °C for two hours. The content of CBL was determined by comparing the sample curve with the standard curve, which was obtained from CBL- BSA standard.^7^

### Statistical analysis

SPSS 22.0 software (IBM Corporation, Armonk, NY, USA) was used for data analysis and processing. The measured data that exhibited normal distribution were expressed as mean ± Standard Deviation (*χ* ± S.E.). An independent-sample *t*-test or paired *t*-test was performed for comparing the two groups. The measured data that exhibited non-normal distribution were expressed as M (P25, P75). The Mann-Whitney *U* test was conducted for comparing two independent samples. A correlation between continuous variables was determined by using Spearman’s correlation coefficient. A Receiver Operating Characteristic (ROC) curve was used to analyze the diagnostic value of HCit for SK. Moreover, the youden index of the ROC curve was also used to determine the critical values of accuracy, sensitivity, and specificity. In this experiment, p < 0.05 indicated a statistically significant difference.

## Results

The expressions of HCit in healthy control skin increased with the age by immunohistochemistry

**Figure 1:**
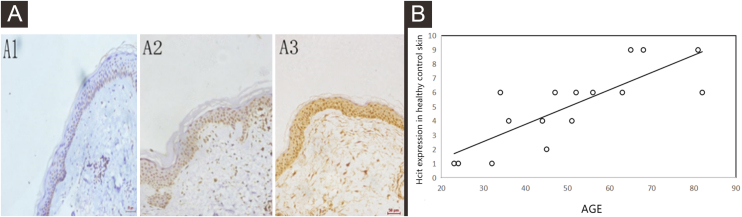
Expressions of HCit in healthy control skin at different ages. 10 high power microscope fields (400×) were randomly selected for observing each section, and scoring was performed as per staining intensity and the percentage of positive cells in the epidermis. The percentage of positive cells was scored as 0‒4 points: < 5%, 0; point 5%‒25%, 1; point 25%‒50%, 2, points 50%‒75%, 3, points ≥ 75%, 4 points. The staining intensity was scored as 0‒3 points: non-staining, 0; Light yellow, 1; Brownish yellow, 2; Chocolate brown, 3. The above-mentioned points were multiplied together to obtain the final score, and higher the score, stronger was the positive expression of HCit. The measured data was expressed as mean ± standard deviation (*χ* ± s). (A) Representative images of immunohistochemistry of different ages (A1: 32 years old, A2: 51-years old, A3: 81-years old). (B) Representative the correlation plot between age and immunohistochemical score of healthy control skin at different ages. the correlation coefficient was 0.806, p = 0.0002. Data were analyzed using Pearson. HCit, Homocitrulline.

By performing immunohistochemistry of healthy control skin tissues, the authors found that the nuclei of epidermal keratinocytes were stained yellowish-brown, and the positive staining intensity increased with the age of subjects (Fig. 1A). Moreover, the immunohistochemical score was correlated to the age of subjects, the correlation coefficient was 0.806, p = 0.0002 (Fig. 1B).

The expression of HCit in SK lesions was higher than that in healthy control skin by immunohistochemistry.

**Figure 2:**
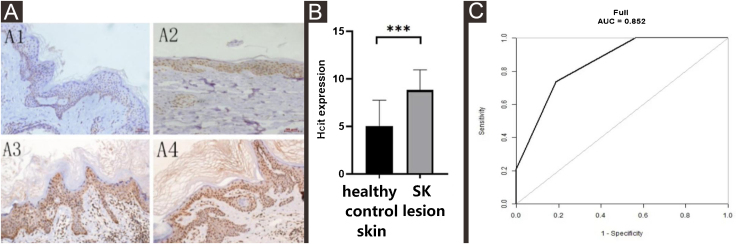
Expression levels of HCit in healthy control skin and lesion of SK. 10 high power microscope fields (400×) were randomly selected for observing each section, and scoring was performed as per staining intensity and the percentage of positive cells in the epidermis. The percentage of positive cells was scored as 0‒4 points: < 5%, 0, point 5%‒25%, 1; point 25%‒50%, 2; points 50%‒75%, 3; points ≥ 75%, 4 points. The staining intensity was scored as 0‒3 points: non-staining 0, Light yellow 1, Brownish yellow 2, Chocolate brown 3. The above-mentioned points were multiplied together to obtain the final score, and higher the score, stronger was the positive expression of HCit. The measured data was expressed as mean ± standard deviation (*χ* ± s). (A) Representative images of Immunohistochemistry of healthy control skin and SK lesion (A1, A3 represent Immunohistochemistry images of healthy control skin and SK lesion at 51 years old respectively. A2, A4 represent Immunohistochemistry images of healthy control skin and SK lesion at 80-years old respectively). (B) Each Bar represents the mean average of the Immunohistochemistry score ± S.E. and data compared between two grops using Student’s *U*-tests (***p < 0.001). (C) Representative image of ROC curve of HCit for SK diagnosis. HCit, Homocitrulline; SK, Seborrheic Keratosis; ROC curve, Receiver Operating Characteristic curve.

The expression of HCit in SK lesions was higher than that in healthy control skin (Z = −3.703, p = 0.0002) (Table 2, Fig. 2A and B). The expression of HCit in skin lesions of SK patients was not correlated to the age of patients (The correlation coefficient was 0.165, p = 0.499). The ROC curve of HCit predicted the diagnosis of SK, the AUC value of this curve was 0.8520, the cut-off value was 0.6119, the sensitivity value was 0.7368, and the specificity value was 0.8125 (Fig. 2C).

**Table 2:** Comparison of expression of HCit between SK lesion and healthy control skin, SK lesion and unaffected skin.

Group	Cases	Score of HCit expression	p-Value
SK lesion	19	9 (6, 9)	
Unaffected skin	19	9 (9, 9)	0.056
Healthy control skin	16	6 (3.5, 6)	0.0002

The unaffected skin was collected in 0.5 centimeters from SK skin.

HCit, Homocitrulline; SK, Seborrheic Keratosis.

There was no significant difference in the expression of HCit between SK lesions and unaffected skin by immunohistochemistry.

**Figure 3:**
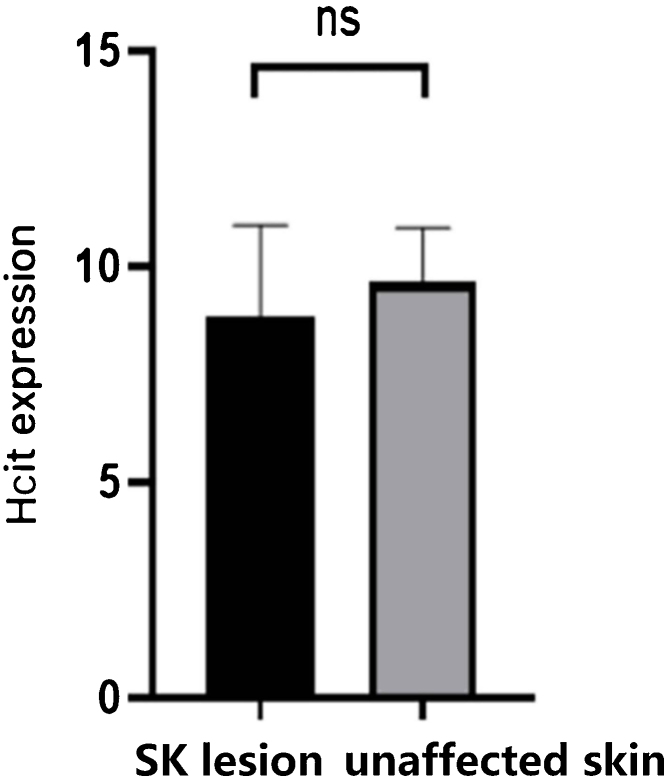
Expression of HCit in SK lesion and unaffected skin. The score was performed as per staining intensity and the percentage of positive cells in the epidermis (method as Fig. 1), The measured data was expressed as mean ± standard deviation (*χ* ± s), Each Bar represents the mean average of the Immunohistochemistry score ± S.E. Data were analyzed using Student’s *t*-tests (ns, no significant difference). HCit, Homocitrulline; SK, Seborrheic Keratosis.

As Table 2 and Fig. 3 indicated, there was no significant difference in the expression of HCit in skin lesions and unaffected skin of SK patients (t = −2.041, p = 0.056). The expression of HCit in unaffected skin was not correlated to the age of patients (the correlation coefficient was 0.033, p = 0.894).

The serum level of CBL in the patients of SK was higher than that in healthy subjects by ELISA.

**Figure 4:**
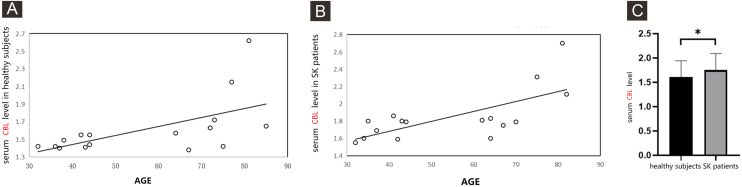
A comparison the serum level of CBL in healthy subjects and in SK patients by ELISA. The supernatants of serum samples from 16 healthy subjects, 16 SK patients were collected for ELISA. (A) Representative the correlation between the serum level of CBL with the age of healthy subjects, the correlation coefficient was 0.5763, p = 0.0032. Data were analyzed using Pearson. (B) Representative the correlation between the serum level of CBL with the age of SK patients, the correlation coefficient was 0.682, p = 0.004). Data were analyzed using Pearson. (C) Each Bar represents the mean average of serum level of CBL ± S.E. with healthy subjects and SK patients. Data were analyzed using *U*-tests (*p < 0.05). CBL, Protein Carbamylation of Lysine; SK, Seborrheic Keratosis; ELISA, Enzyme Linked Immunosorbent Assay.

The serum level of CBL in healthy subjects was correlated with the age of subjects (the correlation coefficient was 0.5763, p = 0.0032) (Fig. 4A). The serum level of CBL in SK patients was also correlated with the age of subjects (the correlation coefficient was 0.682, p = 0.004) (Fig. 4B). In healthy subjects and SK patients of the similar age group, the serum level of CBL were 1.61 ± 0.33 and 1.85 ± 0.29, respectively, there was a statistically significant difference between the two groups (Z = 2.19, p = 0.030) (Fig. 4C).

## Discussion

Skin aging is a lengthy and complex evolutionary process, which progresses gradually with age. Although skin aging has many manifestations, there is no objective index to measure skin aging till date. In this study, the authors found that the contents of HCit (CBL) in the skin and serum of healthy subjects of different ages were positively correlated with the age of subjects. Moreover, the expression of HCit in SK patients was higher than that in healthy subjects. The content of CBL in the peripheral serum of SK patients was higher than that in healthy subjects, HCit can be used to predict the diagnosis of SK, implying that HCit may be one of the potential biomarkers of skin aging.

Carbamylation reaction is a process in which a cyanate molecule and its structural isomer, that is, isocyanate combine with free functional groups of proteins, polypeptides, or free amino acids.^6^ This reaction usually occurs with lysine ε-amino groups, forming N(6)-Carbamoyl-L-Lysine (CBL): the common name for this compound is Homocitrulline (HCit), which is the most characteristic carbamyl reaction product.^7^ Previous studies have reported that protein carbamylation reaction occurs in patients with chronic diseases, such as chronic kidney disease, atherosclerosis, and rheumatoid arthritis,^8^, ^9^, ^10^ but only a few studies have reported the relationship between protein carbamylation reaction and skin aging. Gorisse et al.^11^ found that the contents of HCit in total skin extract increased significantly with the age of subjects. Moreover, the content of type 1 collagen also increased significantly with the age of subjects: the skin tissues analyzed were of humans, bovine, and rats.

Carracedo et al.^12^ analyzed carbamylated proteins in the peripheral blood of healthy subjects. They found that carbamylated protein levels in peripheral blood were correlated to the age of subjects, the older the age of subjects, the higher the level of HCit. The results of the present study are similar to those of previous studies, which indicates that HCit is correlated to age and aging. In protein carbamylation reaction, the structural and functional properties of proteins and their interaction with cells are altered, leading to protein molecular aging.^17^ Collagen and elastin molecules in the skin have a long half-life period, they contain more lysine and hydroxylysine, which are the preferred targets for carbamylation reactions.^18^ Previous studies have reported that after carbamylation reaction, the tertiary helix structure of type I collagen proteins changed slightly. This impaired the ability of type 1 collagen to transform into normal fibers and also decreased its thermal stability, which will lead to disorder in the extracellular matrix. All these events interfered with the homeostasis of tissues. Furthermore, collagen protein also played a crucial role in structuring connective tissue, it participated in the key event of the host defense mechanism ‒ white blood cell activation. However, carbamylated collagen lost the ability to stimulate neutrophil oxidation through LFA-1 integrin and Focal Adhesion Kinase (P125FAK) signaling pathway, which was known to be involved in the development of inflammation and infection,^19^, ^20^ thus, protein carbamylation reaction causes skin aging changes, such as loose skin, wrinkles, and inflammation.

Many studies have shown that SK is a manifestation of local skin aging.^13^, ^14^, ^15^, ^16^ Besides determining the difference in HCit expression among healthy subjects of different ages, the authors selected SK patients as the study participants. The authors found that the expression of HCit in skin lesion of SK patients was higher than that in healthy control skin, and the content of CBL in the peripheral serum of SK patients was higher than that of healthy subjects, the authors also found that HCit could be a predictor in the diagnosis of SK, which further proved the correlation between HCit and skin aging. The main limitations of this study are the small serum sample size, the authors will enlarge the sample size for further study.

## Conclusion

Seborrheic Keratosis (SK) is a common disease in dermatology, its incidence increases along with increasing age,^4^ it is a manifestation of skin aging. A high expressional level of HCit is correlated with seborrheic keratosis and skin aging, HCit may be one of the potential biomarkers of seborrheic keratosis and skin aging.

## Financial support

This study was supported by the Wu JiePing Clinical Medicine Foundation (320.6750.19089-31) and the Major projects of the Natural Science Foundation of the Jiangsu Higher Education Institutions of China (Grant Nº 18KJA320014).

## Authors’ contributions

Juping Chen: Approval of the final version of the manuscript; Critical literature review; Intellectual participation in propaedeutic and/or therapeutic management of studied cases; Manuscript critical review; Preparation and writing of the manuscript; Study conception and planning.

Jun Liu: Data collection, analysis and interpretation; Preparation and writing of the manuscript; Statistical analysis.

Zheng Wang: Effective participation in research orientation.

Jiandan Xu: Effective participation in research orientation.

Jia Tao: Effective participation in research orientation.

Hualing Li: Approval of the final version of the manuscript; Intellectual participation in propaedeutic and/or therapeutic; Management of studied cases; Manuscript critical review; Study conception and planning.

## Conflict of interest

None declared.
